# Report of *Platythomisus octomaculatus* (C. L. Koch, 1845) and *Platythomisus sudeepi* Biswas, 1977 from India (Araneae, Thomisidae)

**DOI:** 10.3897/BDJ.5.e10294

**Published:** 2017-01-31

**Authors:** Swara Yadav, Vinayak Patil, Vijay Anand Ismavel

**Affiliations:** 1 Zoology Department, Yashavantrao Chavan Institute of Science, Satara, Satara, Maharashtra, India; 2 College of Forestry, Dapoli 415712, District Ratnagiri, Maharashtra, India; 3 Makunda Christian Leprosy and General Hospital, Bazaricherra, District Karimganj, Assam, India

**Keywords:** Crab spider, Taxonomy, morphology, Western Ghats.

## Abstract

**Background:**

The genus *Platythomisus* Doleschall, 1859 presently comprises 13 valid species, nine known from Africa and four from Asia. All *Platythomisus* species are known from females only, except *P. jucundus* Thorell, 1894 and *P. sudeepi* Biswas, 1977 from both sexes and *P. quadrimaculatus* from juvenile. Only, *P. sudeepi* was reported from India.

**New information:**

*Platythomisus octomaculatus* (C. L. Koch, 1845) is recorded after 120 years of its last report; newly recorded from Assam, India which extends its distribution from the previously known localities, Java and Sumatra. *Platythomisus sudeepi* is newly recorded from the Maharashtra State. The variation in the number of abdominal spots on juvenile, sub-adult and adult of *P. octomaculatus* observed during rearing is reported. Although, the species name '*octomaculatus*' suggests eight spots, we observed that the anterior pair of abdominal spots is fused in adults.

## Introduction

During a field survey in the private property of Makunda Christian Leprosy & General Hospital, Karimganj District, Assam, we found a bright yellow thomisid spider with black spots. Based on the literature Koch (1845), Doleschall (1859), Hasselt (1882) and Simon (1895) we were able to identify it as *Platythomisus octomaculatus* (C. L. Koch, 1845). Another pale red coloured female *Platythomisus* with three transverse black bands was collected from Dapoli in the Western Ghats of Maharashtra. Biswas (1977) and Siliwal and Molur (2005) revealed its identity to be *Platythomisus sudeepi* Biswas, 1977.

Koch (1845) described *Thomisus 8-maculatus* based on female from ‘Ostindien’ which is a German term for East Indies (in present day Indonesia), where he stated that the spider has 8 black spots on its dorsum and has also been represented in the diagram. Doleschall (1859) described the genus *Platythomisus* with the description of *Platythomisus phryniformis* from Java. Hasselt (1882) mentioned *Thomisus 8-maculatus* as Platythomisus (Thomisus) octomaculatus for his specimens from Padang, Indonesia. Later, *Platythomisus phryniformis* was synonymized with *P. octomaculatus* by Hasselt (1890). *Platythomisus octomaculatus* is presently known to be distributed in Sumatra and Java (Koch 1845, Doleschall 1859, Hasselt 1882, WorldSpiderCatalog 2016). Given the striking colours of this species, hundreds of photographic records are available on internet, mainly from Singapore, adding to the known distribution.

Biswas (1977) described *Platythomisus sudeepi* based on female from Pollibetta, Coorg, Karnataka. Later, *P. sudeepi* was reported from Castle rock, Karnataka (Bastawade et al. 2004), Thrissur, Kerala (Siliwal and Molur 2005). Very recently, Benjamin et al. (2016) reported this species from Sri Lanka with first description of its male. Presently, *P. sudeepi* is known from the Western Ghats of India and Sri Lanka.

## Materials and methods

Specimens are preserved in 70% alcohol, deposited at the Bombay Natural History Society (BNHS), Mumbai. Specimens were studied under a Leica stereozoom (MZ6) microscope, photographed using mounted Canon Powershot S50 camera, assembled using Combine ZM software and the images were processed with Adobe Photoshop CS5. Measurements were done with Erma stage and ocular micrometer and an accurate scale. Epigyna were cleared in 10% KOH and kept in Polyvinyl Lactophenol (PVLP) gel with Lignin pink stain for seven days before imaging. All measurements are in millimetres; measurements of other specimen of *P. octomaculatus* are provided in parentheses. Map was produced with DIVA-GIS v. 7.5c, with geographical coordinates obtained from Google Earth.

### Abbreviations used

Depository: BNHS - Bombay Natural History Society, Mumbai, India (Curator-Rahul Khot); NHMW- Natural History Museum, Vienna, Austria (Curator- Christoph Hörweg).

## Taxon treatments

### Platythomisus octomaculatus

(C. L. Koch, 1845)

#### Materials

- **Type status:** Other material. **Occurrence:** recordedBy: Antina Pasyad; individualCount: 1; sex: female; lifeStage: adult; **Taxon:** scientificName: *Platythomisus octomaculatus* (C. L. Koch, 1845); kingdom: Animalia; phylum: Arthropoda; class: Arachnida; family: Thomisidae; genus: Platythomisus; taxonRank: species; taxonomicStatus: accepted; **Location:** locationID: Makunda Christian Hospital Campus; continent: Asia; country: India; countryCode: IN; stateProvince: Assam; verbatimLocality: Makunda Christian Hospital Campus, Karimganj District; verbatimCoordinateSystem: decimal degrees; decimalLatitude: 24.433; decimalLongitude: 92.326; **Identification:** identifiedBy: Siddharth Kulkarni; **Event:** year: 2016; month: 4; day: 19; **Record Level:** type: PhysicalObject; institutionID: BNHS; collectionID: Sp; collectionCode: Arachnida; basisOfRecord: PreservedSpecimen
- **Type status:** Other material. **Occurrence:** recordedBy: Rejoice Gassah; individualCount: 1; sex: female; lifeStage: adult; **Taxon:** scientificName: *Platythomisus octomaculatus* (C. L. Koch, 1845); kingdom: Animalia; phylum: Arthropoda; class: Arachnida; family: Thomisidae; genus: Platythomisus; taxonRank: species; taxonomicStatus: accepted; **Location:** locationID: Makunda Christian Hospital Campus; continent: Asia; country: India; countryCode: IN; stateProvince: Assam; verbatimLocality: Makunda Christian Hospital Campus, Karimganj District; verbatimCoordinateSystem: decimal degrees; decimalLatitude: 24.433; decimalLongitude: 92.326; **Identification:** identifiedBy: Siddharth Kulkarni; **Event:** year: 2016; month: 6; day: 30; **Record Level:** type: PhysicalObject; institutionID: BNHS; collectionID: Sp; collectionCode: Arachnida; basisOfRecord: PreservedSpecimen

#### Description

Total length- 10.7 (14.2) mm; Carapace width- 4.9 (5.2) mm; Ophisthosoma widest- 5.1 (5.9) mm; Leg I- 19.8 mm; Leg II- 21 mm; Leg III- 11.3 mm; Leg IV- 12.5 mm.

Medium sized-spider. Carapace pale orange with two large and one small paired black spots dorsally; larger spots on ocular region and near fovea, smaller spots on lateral sides along mid-length of carapace. Chelicerae base, fangs, palpal patella to claws, black. Legs yellow (become paler in ethanol), tibia to tarsus I, II and metatarsus, tarsus III, IV black. Abdomen yellow with three pairs of large spots arched by a large spot anteriorly (Fig. 1a); ventrally black patch narrowed near spinnerets, laterally yellow; spinnerets black (Fig. 1b).

Cephalothorax without hair, slightly convex dorsally, abdomen dorso-ventrally flat. Carapace narrow anteriorly, wider posteriorly, surface covered with inconspicuous tubercles. Sternum sub-triangular, maxillae, labium oval, rebordered. Legs slender, II>I>IV>III. Abdomen grossly oval, anteriorly truncate, posteriorly narrow and wrinkled along margins.

Spermathecae kidney-shaped, sclerotized, with folds, narrow at base, closer to each other at apex (Fig. 1c); ventrally, epigyne with weakly sclerotized margins of round hood, open at spermathecae second half-length (Fig. 1d).

##### Remarks

**Figure 2.**
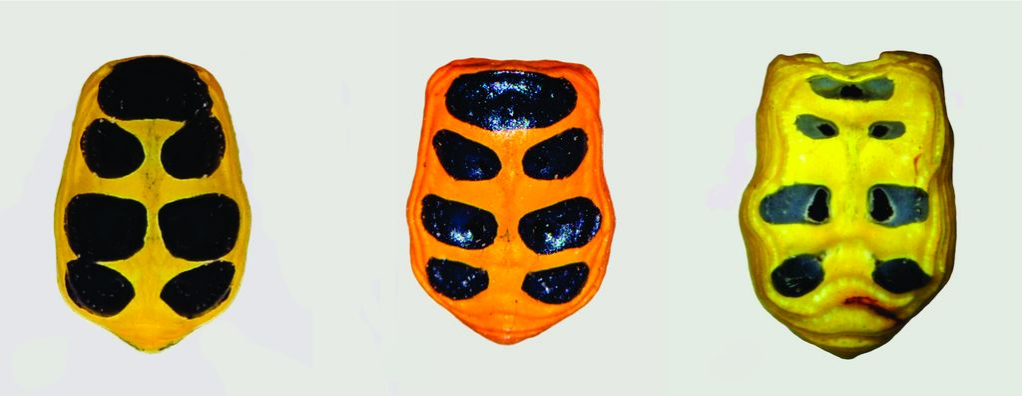
*Platythomisus octomaculatus*, abdominal spots (left to right) before egg laying, just after egg laying, late after egg laying

Koch (1845) most likely named the species as ‘*octomaculatus*’ for the eight apparent abdominal spots as shown in his illustration. Doleschall (1859) described his specimen of *P. octomaculatus* had seven round black spots on the dorsum of which the first one is unpaired. Hasselt (1882) mentioned that his specimens match with that of Doleschall’s with the seven spots on abdomen; as also seen in our specimens. Eight spots are present in the sub-adult female of *P. octomaculatus*, of which the first pair of spots is merged in the adults as observed in Singaporean specimens (pers comm. David Court, Singapore). It was also observed that the size of these seven spots was variable before and after egg laying (Fig. 2). The young ones emerging from the egg case of *P. octomaculatus* (Fig. 3a) have only two abdominal spots at early stages (Fig. 3b). In the specimens from Java and Fort de Kock, Sumatra (deposited at the NHMW), Singapore and India, the position of spots on carapace and abdomen seems to be constant, however their size is variable.

#### Distribution

Known from localities in Java, Sumatra and India (see map, see Introduction).

### Platythomisus sudeepi

Biswas, 1977

#### Materials

- **Type status:** Other material. **Occurrence:** recordedBy: S. B. Shelke; individualCount: 1; sex: female; **Taxon:** scientificName: *Platythomisus sudeepi* Biswas, 1977; kingdom: Animalia; phylum: Arthropoda; class: Arachnida; family: Thomisidae; genus: Platythomisus; taxonRank: species; taxonomicStatus: accepted; **Location:** continent: Asia; country: India; countryCode: IN; stateProvince: Maharashtra; county: Ratnagiri; locality: Dapoli; decimalLatitude: 17.747; decimalLongitude: 73.182; **Identification:** identifiedBy: Vinayak Patil; **Event:** year: 2013; month: 10; day: 15; **Record Level:** type: PhysicalObject; institutionID: BNHS; collectionID: Sp; basisOfRecord: PreservedSpecimen

#### Description

Total length- 8.5 mm; Carapace width- 3.66 mm; Ophisthosoma widest- 3.86 mm; Leg I- 16.45 mm; Leg II- 17.45 mm; Leg III- 11.00 mm; Leg IV- 11.38 mm.

Medium-sized spider. Carapace reddish orange at life, brownish in alcohol, with four conspicuous round black spots forming trapezium, wider posteriorly. All eyes except AME situated on anterior spots. Legs slender, patella to tarsus black, except metatarsus which is yellowish with black dorsal line. Femur I black, proximal half of II, III red, rest black, IV entirely red. Chelicerae short, pale orange; palps black. Maxillae and labium black distally. Abdomen reddish orange with three black transverse bands, the posterior-most being the thickest and widest (Fig. 4a); ventrally black patch upto the spinnerets, laterally red. Spinnerets black (Fig. 4b).

Cephalothorax without hair, glabrous, slightly convex dorsally, narrow anteriorly, wider posteriorly. Sternum sub-triangular, maxillae, labium oval, rebordered. Legs slender, II>I>IV>III. Abdomen dorso-ventrally flat, roughly pentagonal, longer than wide, wider posteriorly, extending beyond dorsum, wrinkled along margins.

Spermathecae oblong, sclerotized, with folds, closer to each other at apex (Fig. 4c); ventrally, epigyne with inverted U-shaped, sclerotized hood ventrally located at spermathecae apex (Fig. 4d).

##### Remarks

The geographical coordinates (14°28' N, 74°20' E) for location of Castle rock, North Kanara, Karnataka, near Goa border provided for this species in Bastawade et al. (2004) points in the Arabian Sea. The precise coordinates are 15° 23' 39'' N, 74° 20' 11'' E.

The epigynum illustrated in Benjamin et al. (2016) shows kidney-shaped spermathecae versus oblong shape in our specimen, although it position and shape is similar. Our low sample size did not allow studying the range of variation in this species and needs further examination.

#### Distribution

**Figure 5.**
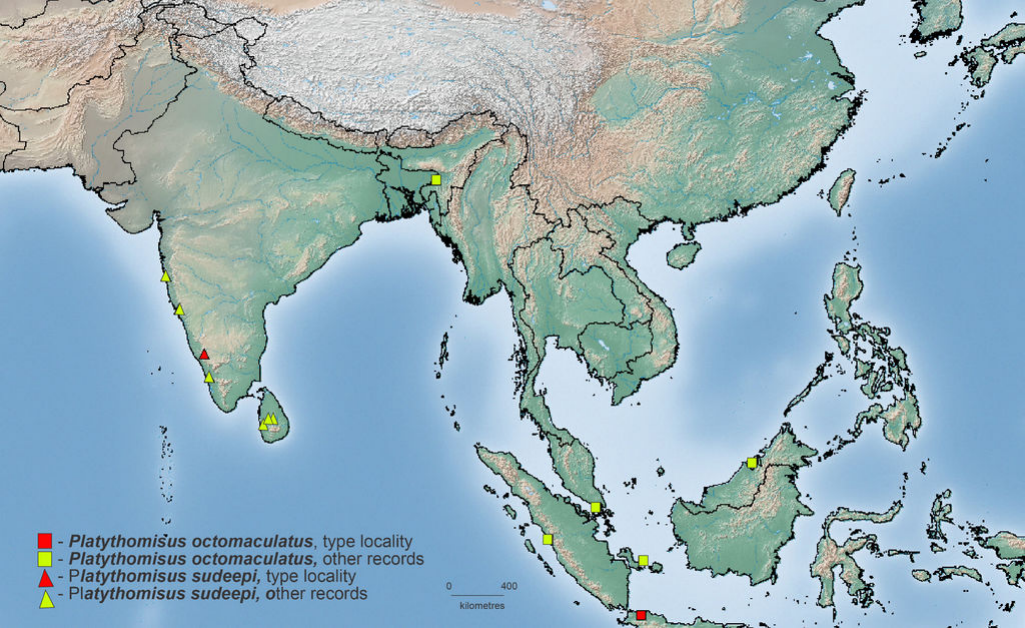
Map showing distribution of *Platythomisus octomaculatus* and *Platythomisus sudeepi*.

Known from several localities in the Western Ghats of India and Sri Lanka (Fig. 5, see Introduction).

## Supplementary Material

XML Treatment for Platythomisus octomaculatus

XML Treatment for Platythomisus sudeepi

## Figures and Tables

**Figure 1a.**
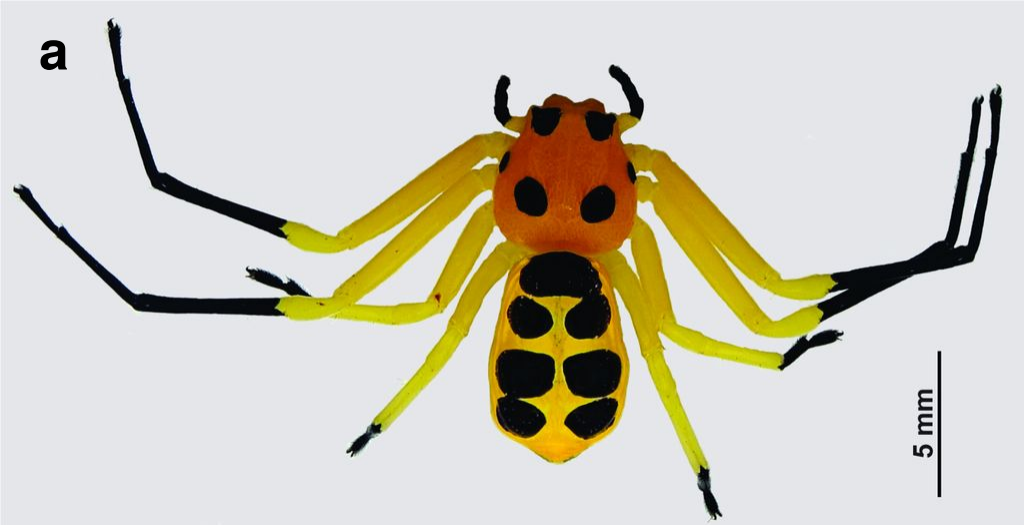
habitus, dorsal view

**Figure 1b.**
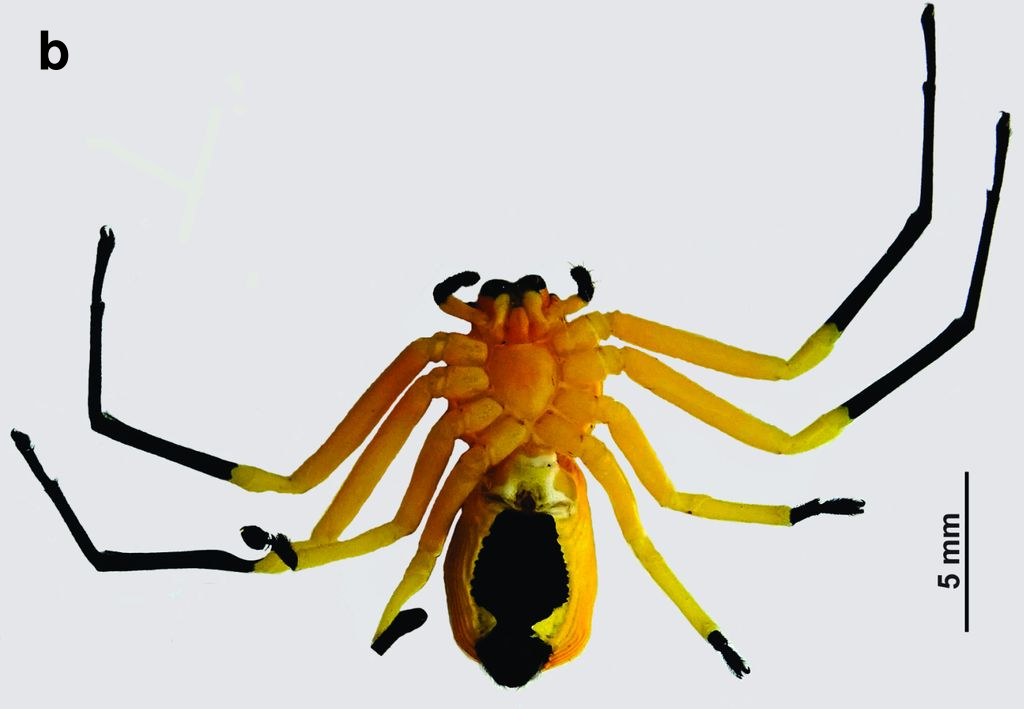
habitus, ventral view

**Figure 1c.**
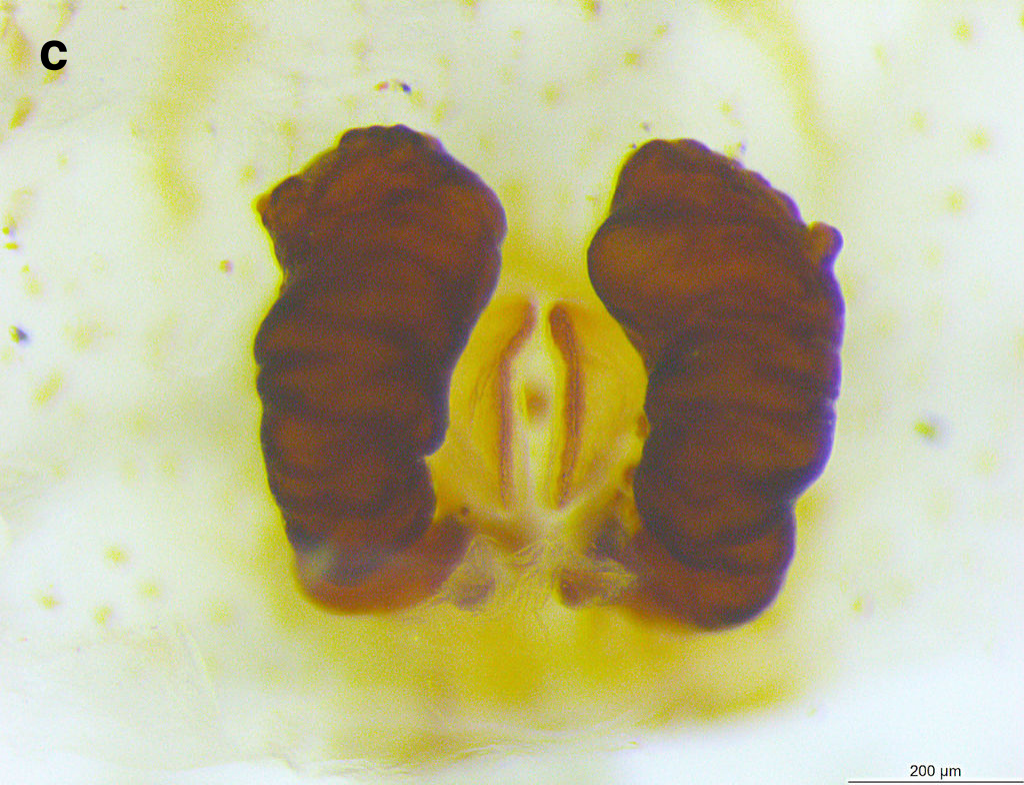
epigynum , dorsal view

**Figure 1d.**
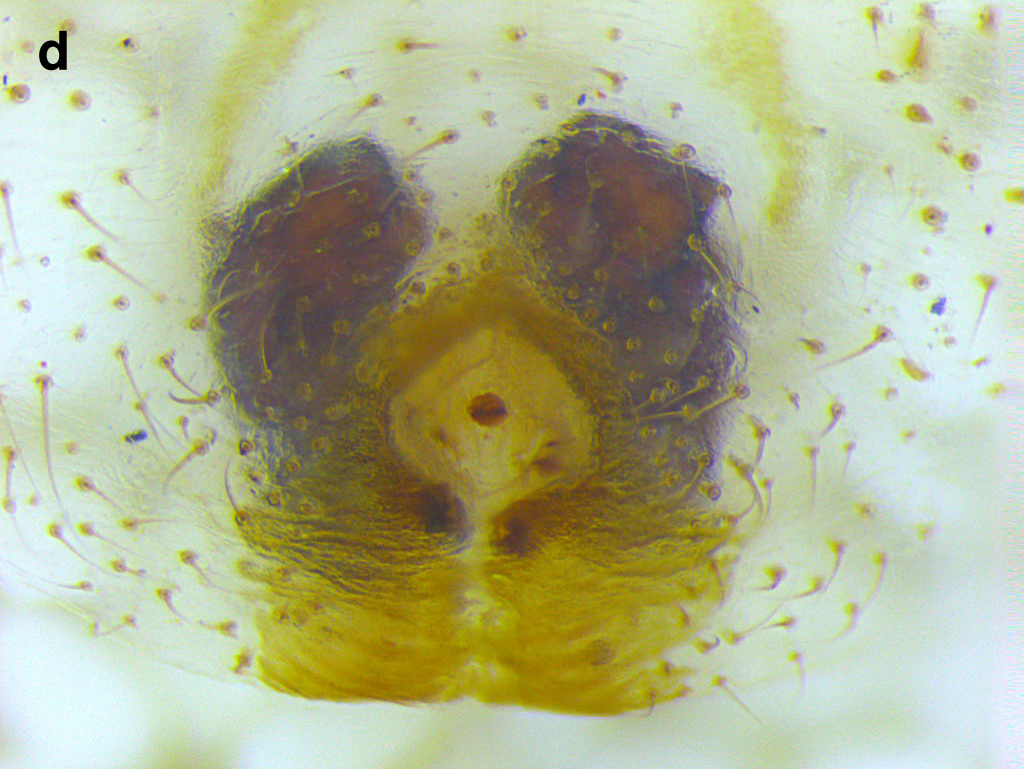
epigynum, ventral view

**Figure 3a.**
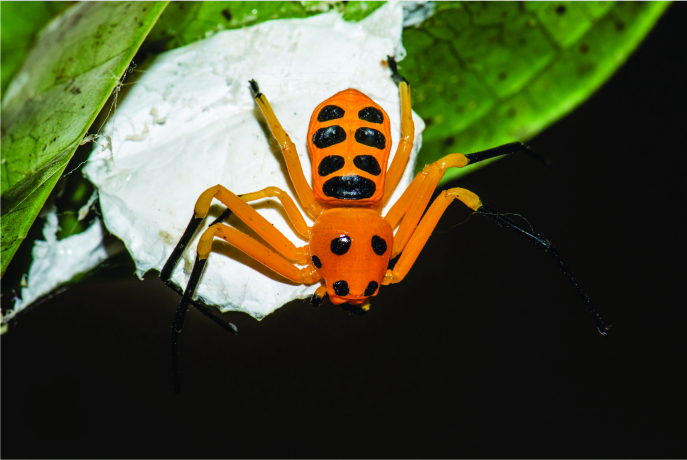
Female guarding egg sac

**Figure 3b.**
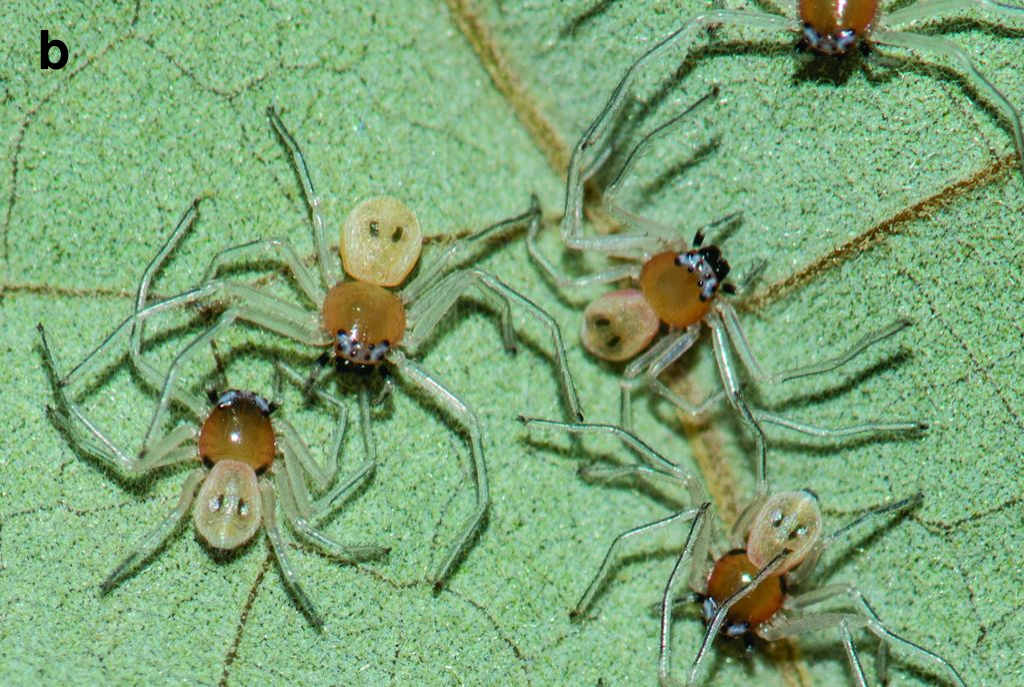
spiderlings

**Figure 4a.**
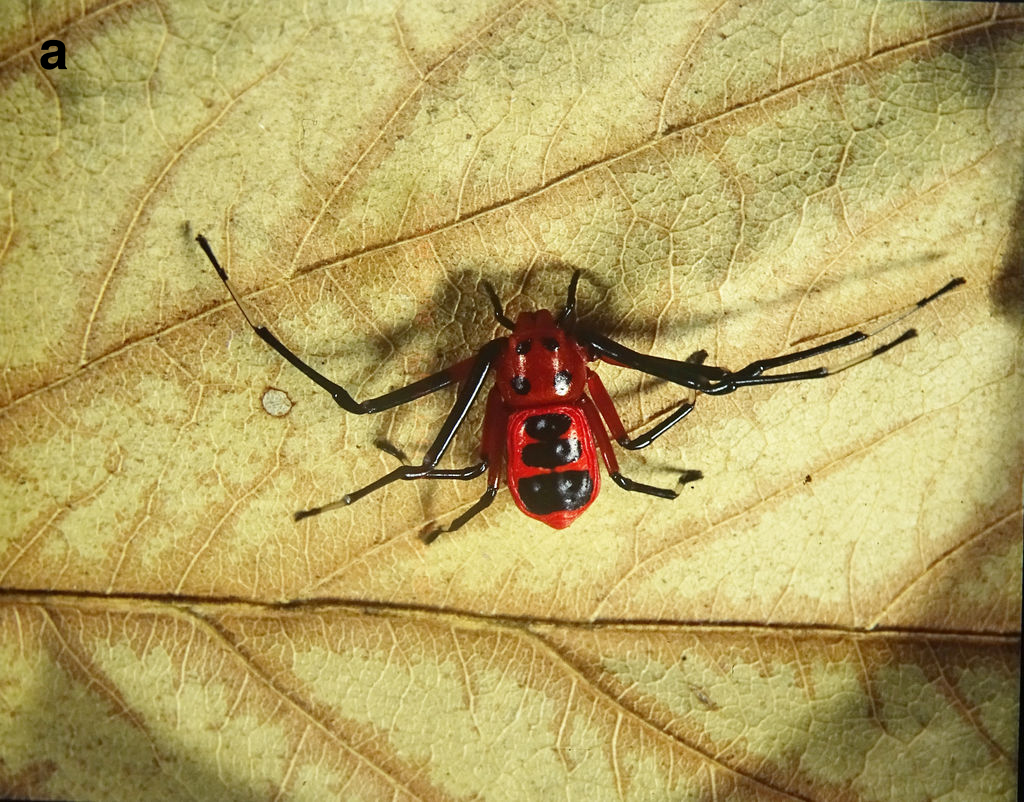
habitus, dorsal view

**Figure 4b.**
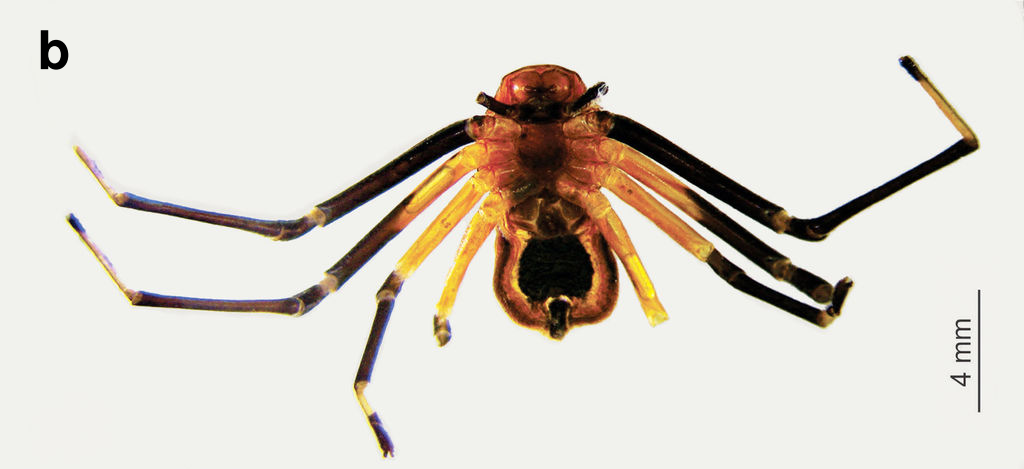
habitus, ventral view

**Figure 4c.**
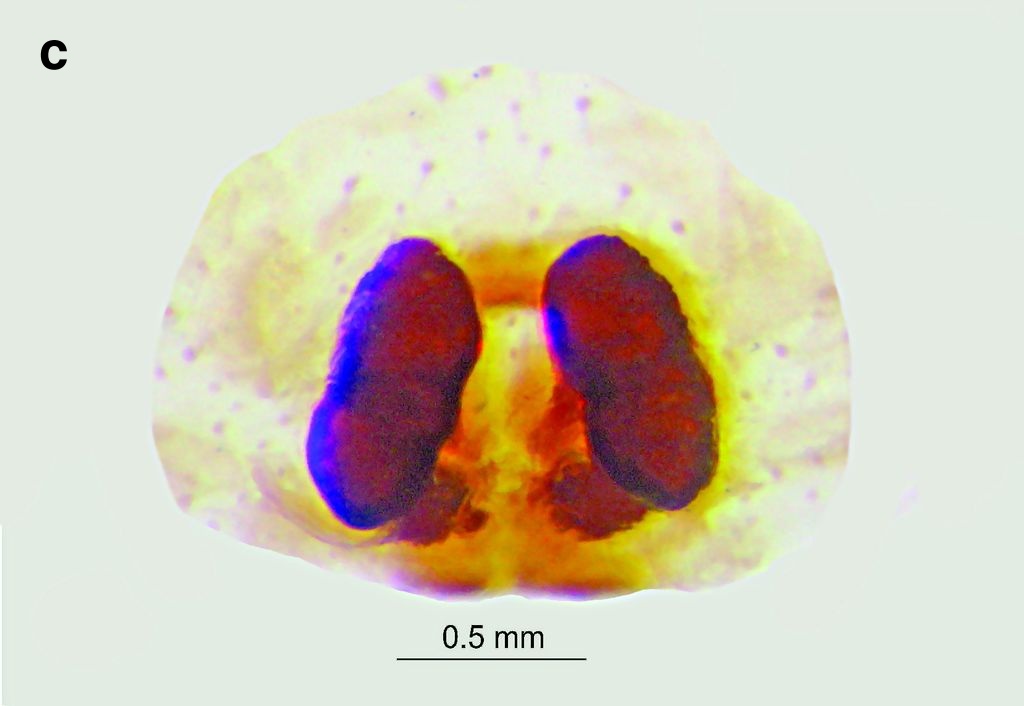
epigynum, dorsal view

**Figure 4d.**
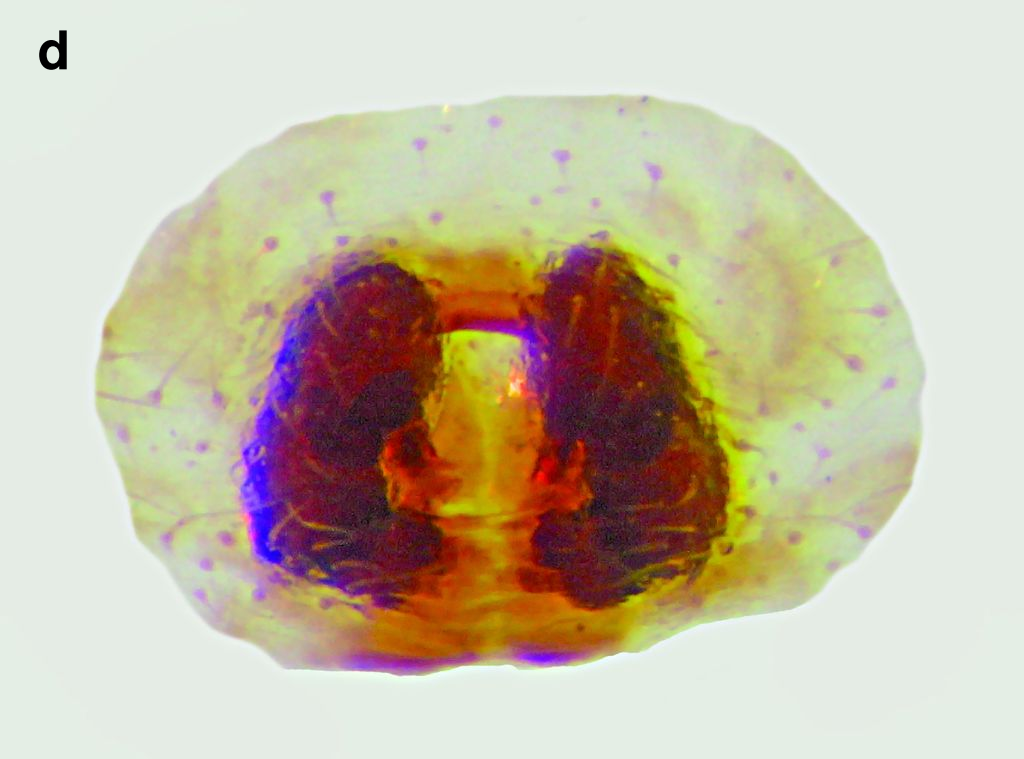
epigynum, ventral view
